# Do different intraoperative glove practices reduce surgical site infections? A systematic review

**DOI:** 10.1186/2047-2994-4-S1-O30

**Published:** 2015-06-16

**Authors:** C Landelle, Z Kubilay, N Damani, T Allen, SL Gans, D Pittet, B Allegranzi

**Affiliations:** 1 Infection Control Program, University of Geneva Hospitals, Geneva, Switzerland; 2 WHO Service Delivery Safety, Clean Care is Safer Care, WHO, Geneva, Switzerland

## Introduction

The invasive nature of surgery carries high risk for the transfer of pathogens responsible for surgical site infections (SSI). This risk can be reduced by using protective barriers such as sterile gloves; however, gloving practices vary among different surgical specialties and countries.

## Objectives

To determine whether double gloving *vs* single gloving, changing gloves during the operation *vs* retaining gloves, and using specific type of gloves reduce SSI rates.

## Methods

We conducted a systematic literature review and searched PubMed, EMBASE, CINAHL, Cochrane CENTRAL, WHO Global Index Medicus, and reference lists of relevant papers for articles published from 1990 to 24/04/2014 in English, Spanish and French. Studies investigating the impact on SSI of the above mentioned interventions related to surgical glove use in patients undergoing surgery were selected.

## Results

The search yielded 1049 articles and 7 were selected. Two studies comparing double gloving *vs* single gloving were identified. A retrospective study including 863 surgical patients showed significantly higher cerebrospinal fluid shunt infection rate in the single-gloved group compared to the double-gloved group. The second nonrandomized, "before/after" study found no significant difference in wound sepsis rates after 200 hernia repairs between the double *vs* single-gloved group. Three randomized control trials (RCT) comparing changing surgical gloves *vs* retaining gloves in obstetrics were identified; no reduction of post-cesarean wound infections and/or endometritis following glove change after delivery of the placenta or the fetus was found. Finally, 2 RCTs compared 3 types of gloves in orthopedic surgery: latex gloves with cotton-cloth outer gloves or latex gloves with outer "orthopedic" gloves or repel cloth gloves between 2 pairs of latex gloves *vs* 2 pairs of latex gloves; no SSI was reported in these trials in either group.

## Conclusion

The available evidence to assess the effect of wearing additional gloves, intraoperative glove change or type of gloves on SSI rates is very limited and of low-quality. Our findings indicate the need for RCTs on this topic.

## Disclosure of interest

None declared.

